# Evaluation of zinc oxide nanocomposite with *Aloe vera* gel for packaging of chicken fillet against *Salmonella typhi* and *Salmonella para typhi* A

**DOI:** 10.1002/fsn3.3528

**Published:** 2023-06-23

**Authors:** Mohammad Mehdi Soltan Dallal, Samira Karimaei, Maryam Hajighasem, Seyed Jamal Hashemi, Abbas Rahimi Foroushani, Mahmoud Ghazi‐Khansari, Alireza Partoazar

**Affiliations:** ^1^ Department of Pathobiology, School of Public Health Tehran University of Medical Sciences Tehran Iran; ^2^ Food Microbiology Research Center Tehran University of Medical Sciences Tehran Iran; ^3^ Division of Food Microbiology, Department of Pathobiology, School of Public Health Tehran University of Medical Sciences Tehran Iran; ^4^ Department of Parasitology and Mycology, School of Public Health Tehran University of Medical Sciences Tehran Iran; ^5^ Department of Epidemiology and Biostatistics, School of Public Health Tehran University of Medical Sciences Tehran Iran; ^6^ Department of Pharmacology, School of Medicine Tehran University of Medical Sciences Tehran Iran; ^7^ Experimental Medicine Research Center Tehran University of Medical Sciences Tehran Iran

**Keywords:** antibacterial activity, food packaging, food quality, nanocomposite, plant extracts, toxicology

## Abstract

The growing demand for high food quality has been encouraging researchers in the food industry to apply biodegradable nanocomposites, which provide new opportunities and challenges for the advance of nanomaterials in the food industry. The objective of this study was to estimate the antibacterial activity and cytotoxicity effects of zinc oxide nanocomposite/zeolite (c/Zeo) with *Aloe vera* gel (AG) and its effect on the shelf life of chicken meat. The ZnONPs/Zeo was assessed using X‐ray fluorescence (XRF) and field emission scanning electron microscopy (FE‐SEM) analyses. The cytotoxicity effect of ZnONPs/Zeo was assessed by MTT assay. Then, the minimum inhibitory concentrations (MIC) and minimum bactericidal concentration (MBC) of ZnONPs/Zeo and ZnONPs/Zeo‐AG against *Salmonella typhi* and *Salmonella para typhi* A were investigated. Also, the preservative effect of nanocomposites on chicken fillets was evaluated. The results showed that these nanocomposites have the least cytotoxicity effect, resulting in good biocompatibility with the host. The MIC and MBC values of ZnONPs/Zeo‐AG were lower than the ZnONPs/Zeo against *S. typhi* and *S. paratyphi* A. Both ZnONPs/Zeo‐AG and ZnONPs/Zeo caused a significant decrease in the bacterial count of the chicken fillets. So, by spraying on meat, the number of bacteria presented a sharper decline as compared with the control group, resulting in an approximately 3.3 and 3‐log_10_ reduction over 48 h in the ZnONPs/Zeo‐AG and ZnONPs/Zeo treatment samples, respectively. In conclusion, antimicrobial packaging with ZnONPs containing *A. vera* is a beneficial solution for preserving and improving the quality, safety, and shelf life of fresh meat products.

## Graphical abstract

The objective of this study was to estimate the antibacterial activity and cytotoxicity effects of zinc oxide nanocomposite/zeolite (ZnONC/Zeo) with *Aloe vera* gel (AG) and its effect on the shelf life of chicken meat.

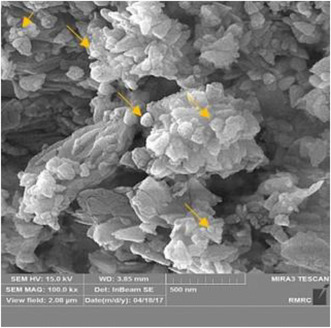

## 1 INTRODUCTION

Appropriate food packaging prevents food degradation by microbial contamination. Hence, nanotechnology is used to meet the consumers' requirements to improve food quality by using antimicrobial agents to prolong the shelf life of foods under storage and delivery conditions (Youssef et al., 2016). Food industries have got to pay more attention to selecting which biodegradable packaging material is more appropriate for their food products. Also, with the increasing request for stability and environmental safety, a rising amount of studies have been concentrated on expanding food packaging materials that can rapidly degrade and entirely mineralize into rather safe products (Ahmadi et al., 2020).

*Aloe vera* has numerous pharmacological properties due to its various potentially bioactive components (Jangra et al., 2022). The main ingredients of aloe consist of anthraquinones, polysaccharides, lectin, alkylbenzenes, dehydroabietic acid, saponin, salicylic acid, and lignin (Moghaddam et al., 2018). Aloin is one of the most active anthraquinone glycosides of aloe latex. It shows an enhanced pharmacological effect because of its facile entrance into the target cells (Arbab et al., 2021). Moreover, *A. vera* displays many pharmacological activities, such as anti‐inflammatory, anti‐ulcer, antitumor, anti‐asthmatic, antidiabetic, and antimicrobial properties (Jangra et al., 2022).

Metal nanoparticles are used in the food industry due to their antimicrobial attributes, principally to cover food processing equipment or in food packaging to reduce foodborne diseases and food decay (Youssef et al., 2016). Zinc oxide NPs (ZnO NPs) is a metal nanoparticle which has attracted much attention due to their unique morphology, and antimicrobial activity against both Gram‐positive and Gram‐negative bacteria (Babapour et al., 2021). Besides, ZnO NPs have an eco‐friendly relationship with the environment and easy preparation. ZnO is approved by the Food and Drug Administration (FDA) as a safe food additive (Tamimi et al., 2021).

The antibacterial effect of ZnO NPs is related to interactions between them and cell membrane compounds, such as the bonding with amino acids, induction of reactive oxygen species (ROS) production, and membrane depolarization (Verma et al., 2018). Furthermore, nanofabrication has a challenge with aggregations, probable cytotoxicity, and expensive material (Razavi et al., 2018). Hereon, nanomaterials as a complex with zeolites will dominate the problems of translational research (Alswat et al., 2016). Even though the study of the biological use of zeolites is a novel field, it is gradually becoming an attractive area. Besides the harmless properties of zeolites, they can be widely used in the near future due to their reversible binding to minor molecules and their capacity of acting similarly to metalloenzymes and regulating immune response (Demirci et al., 2014). Also, nano zinc oxide‐doped zeolite has a major antibacterial potential (Alswat et al., 2016).

Microbial contamination is an important problem effective on food (exclusively meats) resulting in economic fatalities, quality decreases, and diminished product shelf life (Clarke et al., 2016). *Salmonella* spp. is the main common foodborne bacteria in animal‐source foods (ASF). Two species of *Salmonella* associated with ASF were recognized, including *S. enterica* and *S. bongori*. *S. enterica* is related to human salmonellosis (Rortana et al., 2021). *Salmonella* spp. may contaminate fresh meat during butchering or processing, transporting, storing, and selling at the markets (Rortana et al., 2021). These bacteria are generally distributed in the environment, mostly in livestock, such as chickens, and pigs, which colonization may be subclinical and difficult to diagnose before slaughter, then can contaminate carcasses and make humans sick through consumption (Koh et al., 2022). Among the different meats, chicken is broadly consumed because of its low‐fat, nourishing, and comparatively low value (Azlin‐Hasim et al., 2016). This meat is also susceptible to deterioration due to its protein ingredients, besides appropriate pH, permitting the growth of microbes (Takma & Korel, 2019).

Green packaging development can reduce adverse environmental influences by using biodegradable materials, plant extracts, and nanomaterials (Han et al., 2018). Many types of research have been reported on nanomaterials applications in food packaging, but the utmost materials are in the phase of possibility. The use of these materials in food packaging should be approved for their safety because there are public concerns about the possible adverse effects of these materials migrating into food environments (Cwiek‐Ludwicka & Ludwicki, 2017). So, the toxicity assessment of nanomaterials in the food intake of humans is a significant study emphasis.

The present work is a stage toward using bionanocomposites as food packaging supplies. We aimed to investigate the cytotoxicity effect of zinc oxide nanocomposite with *A. vera* gel (AG) on the caco‐2 cell line and to determine their effects on the shelf life of chicken meat.

## 2 MATERIALS AND METHODS

### 2.1 Materials

Zn(CH_3_CO_2_)2·2H_2_O and zeolite powder were purchased from Sigma Chemical Co. Caco‐2 cells were purchased from the National Cell Bank of Iran (Pasteur Institute of Iran, Tehran). Microbial strains, including *Salmonella typhi* and *Salmonella paratyphi* A were provided from our previous study (Nadi et al., 2020). All other chemicals and culture media used in the present study were of analytical grade and purchased from Merck Co.

### 2.2 Preparation of nano zinc oxide/zeolite

ZnONPs/Zeo has been prepared by a method formerly described by Partoazar et al. (2019) with slight modifications. Briefly, zeolite powder was mixed in deionized water for 1 h, and then the wastewater was filtered by a cellulose paper filter. The wash step was done three times and the following drying was performed at 80°C and was kept away from moisture. To get the Zn^2+^ exchanged zeolites, Zn(CH_3_CO_2_)2·2H_2_O (7 g) and zeolite powder (10 g) were added in 100 mL DW and with continuous stirring at 60°C for 1 h. Next, to create nanoparticles, NaOH 1 M solution was added to the suspension till pH = 12 was reached. After 2 h, the solution was washed extensively with DW by a cellulose filter to remove the residual zinc acetate. The nanoparticles were dried overnight at 80°C and then were calcined for 2 h at 400°C. Moreover, the above procedure measured entirely for ZnO/Ze composite construction without the addition of NaOH solution. Zeolite elementals were evaluated to characterize ZnO percentage by X‐ray fluorescence (XRF, PW2404; Philips) system. The morphology of ZnO nanomaterials was analyzed by a field emission scanning electron microscopy (FE‐SEM, MIRA3 TESCAN) system.

### 2.3 Extraction of the *Aloe vera* gel

*Aloe vera* gel has been extracted using a method previously described by Arsene et al. (2022) with a small modification. *A. vera* leaves were collected and cleaned with distilled water. Thirty grams (30 g) of *A. vera* leaf was weighed and added to sterile distilled water (270 mL) in a flask, and incubated at 25°C in a shaker incubator at 300 rpm for 3 h. Then, the mixture was filtered by using a cellulose paper filter. The *A. vera* extract was collected and placed in Petri dishes formerly weighed and then incubated open at 40°C until relative evaporation of aqueous solutions should be done. The extract was collected when the volume was small enough to store at 4°C.

### 2.4 Determining the cytotoxicity effects of ZnONPs/Zeo and ZnONPs/Zeo‐AG

To assess the cytotoxic effects of ZnONPs/Zeo and ZnONPs/Zeo‐AG on Caco‐2 cells, the MTT assay was performed based on Karimaei et al. (2022) study. Briefly, 5000 cells were seeded to each well of 96‐well plates comprising 200 μL of 10% FBS DMEM. After 24 h incubation and cell attachment, the cells were exposed to various concentrations of ZnONPs/Zeo and ZnONPs/Zeo‐AG, including (0.5, 1, 2, 4, 8, and 16 mg/mL) and the plates were incubated for 24, 48, and 72 h at 37°C. After incubation, the medium of each well was exchanged with 100 μL fresh DMEM without FBS. Then, 10 μL of 3‐(4,5‐dimethylthiazol‐2‐yl)‐2,5‐diphenyltetrazolium bromide (MTT; 5 mg/mL; Sigma‐Aldrich) was added to each well for 4 h. Next, the medium was removed and 100 μL dimethyl sulfoxide (Sigma‐Aldrich) was added for 10 min. The optical density of each well was noted at 570 nm by an Eliza reader (Biorad). Finally, a cell survival of more than 75% was considered non‐cytotoxicity (Jung et al., 2019).

### 2.5 Determination of antibacterial activity

The antibacterial activity of *A. vera* extract, zeolite, ZnONPs/Zeo, and ZnONPs/Zeo‐AG was considered against two Gram‐negative pathogenic bacteria (*S. typhi* and *S. paratyphi* A). These bacteria were provided from our previous study (Nadi et al., 2020). The minimal inhibitory concentration (MIC) was estimated by the microdilution method according to the Clinical and Laboratory Standards Institute (CLSI) (Wayne, 2012). First, any of the above compounds were serially diluted in Mueller‐Hinton broth (MHB), at a volume of 100 μL. Then, the overnight bacterial cultures were diluted to reach a bacterial density of 0.5 McFarland standard. Next, 100 μL of bacterial suspension was added to each well, so resulting in a final volume of 200 μL, with different concentrations of compounds (0.25, 0.5, 1, 2, 4, and 8 mg/mL). The control contained only bacterial suspension. After incubation at 37°C for 24 h, MIC was determined as the lowest concentration at which no visible growth of bacteria compared with the control.

To estimate MBC values, 10 μL of inoculums were taken in sterile conditions from negative wells that display the absence of observable turbidity and transferred onto Trypticase Soy Agar (TSA). After incubation at 37°C for 24 h, the lowest concentration caused the elimination of the tested bacteria, which was considered as MBC.

### 2.6 Chicken samples treatment

The chicken fillet meat was collected from different stores in Tehran. All samples were kept in a cooling box for refrigeration while being transported to the laboratory for testing. Determination of organoleptic properties and kinds of microbial tests containing (mold, yeast, and, all microorganisms), match instructions with National standards (Institute of Standards and Industrial Research of Iran [ISIRI], 1997). Treatment of the chicken fillet was performed at refrigeration temperature by spreading the ZnONPs/Zeo and ZnONPs/Zeo‐AG on all the chicken meat samples in two ways (spray on the meat and spray on the packaging). According to our results obtained from investigating the cytotoxic effects of ZnONPs/Zeo and ZnONPs/Zeo‐AG on Caco‐2 cells, the concentration of 8 mg/mL of these compounds was selected and tested on chicken fillets. The chicken fillet sample by adding distilled water was considered as a control. Storage was done at 4°C for 4 days; microbial features were assessed primarily and then at time points: 2, 4, 24, 48, 72, and 96 h.

### 2.7 Microbial evaluation of chicken fillet after treatment

To perform the microbial analysis, 5 g of chicken fillet samples were sterilely cut from different parts and mixed with 45 mL sterile normal saline, and allowed to soak for 20 min. Then, serial dilution was prepared (10^−1^ to 10^−8^) and 1 mL of the last three dilutions (10^−6^, 10^−7^, 10^−8^) was added to the previously sterilized and cooled TSB medium (15 mL). After mixing the medium with the inoculum, it was incubated at 37°C. Plates containing the culture medium without inoculation samples were considered as a negative control. Plates comprising colonies were counted, and the CFU/g was determined (Ahmadi et al., 2020).

### 2.8 Statistical analysis

Data from independently performed experiments are shown as mean ± standard deviation (SD). Statistical comparisons between individual groups were performed by one‐way analysis of variance (ANOVA) with Tukey's post hoc test using GraphPad Prism8 (GraphPad Software, Inc).

## 3 RESULTS

### 3.1 Composite investigation

**FIGURE 1:**
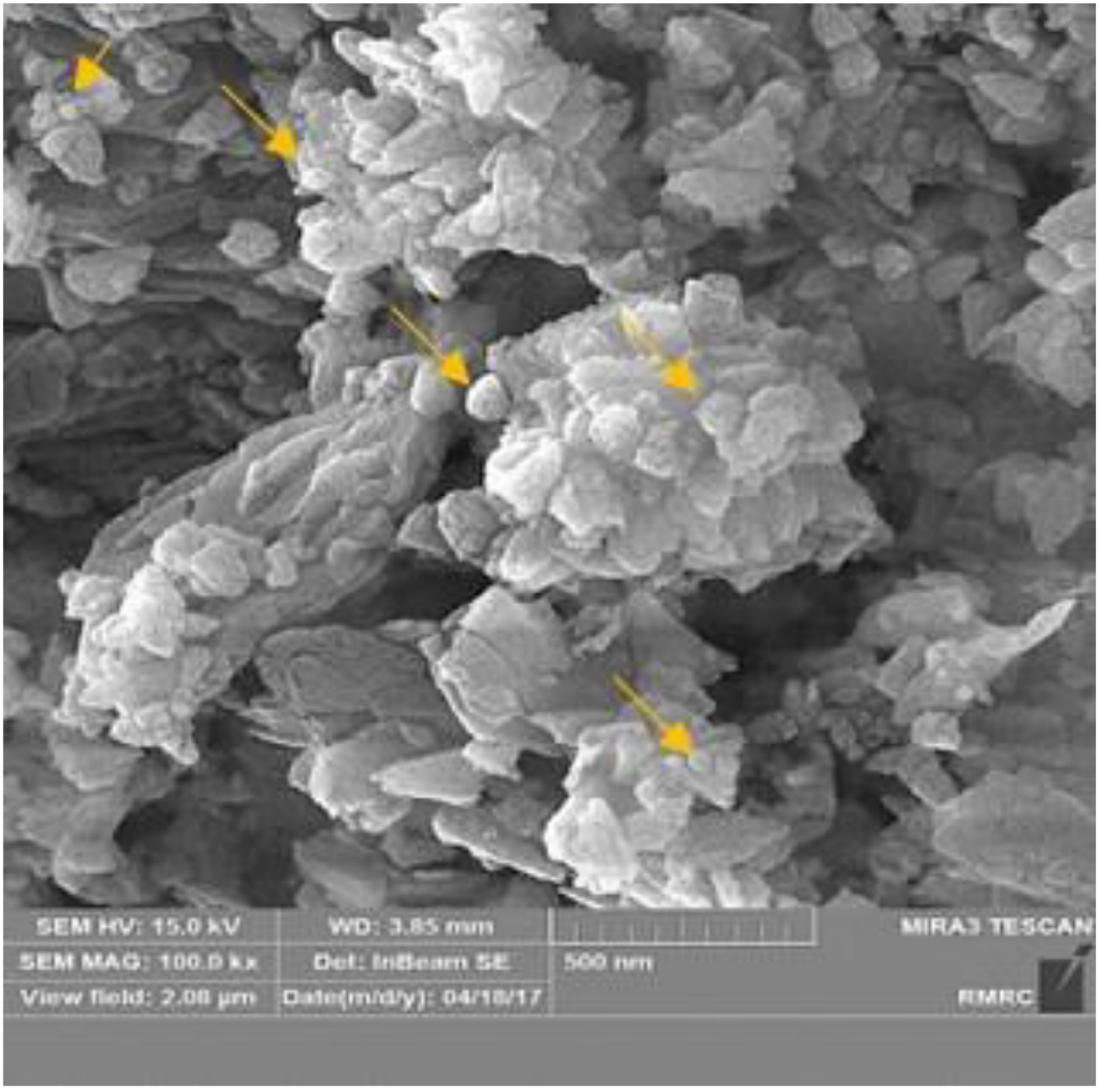
Representative SEM imaging of ZnONPs/Zeo materials. The crystalline forms of Zinc Oxide nanoparticles doped on the surface of the zeolite structure are determined by yellow arrows.

In this study, the XRF technique was used for the evaluation of ZnO percentage in the experimental compounds. The result displays that the sample of NC has 19.34% ZnO among other elements although its quantities were 7.12% and 0.19% for ZnO/Zeo and raw Zeo, respectively (Table 1). Moreover, as shown in Figure 1, high magnification imaging by FE‐SEM showed that crystalline ZnO nanoparticles were formed on the zeolite surfaces.

**TABLE 1:** Elemental analysis for ZnO percentage of the current compounds using the XRF system.

Structure	XRF analysis/(wt. percentage)						
	ZnO	SiO_2_	Al_2_O_3_	CaO	MgO	Fe_2_O_3_	P_2_O_5_
ZnONPs/Zeo	19.34	54.23	8.12	3.67	0.75	0.89	0.08
ZnO/Zeo	7.12	63.91	9.83	4.01	0.99	2.29	0.11
Zeo	0.19	67.12	13.1	4.92	1.7	2.33	0.56

### 3.2 The cytotoxicity effects of ZnONPs/Zeo and ZnONPs/Zeo‐AG on Caco‐2 cells

**FIGURE 2:**
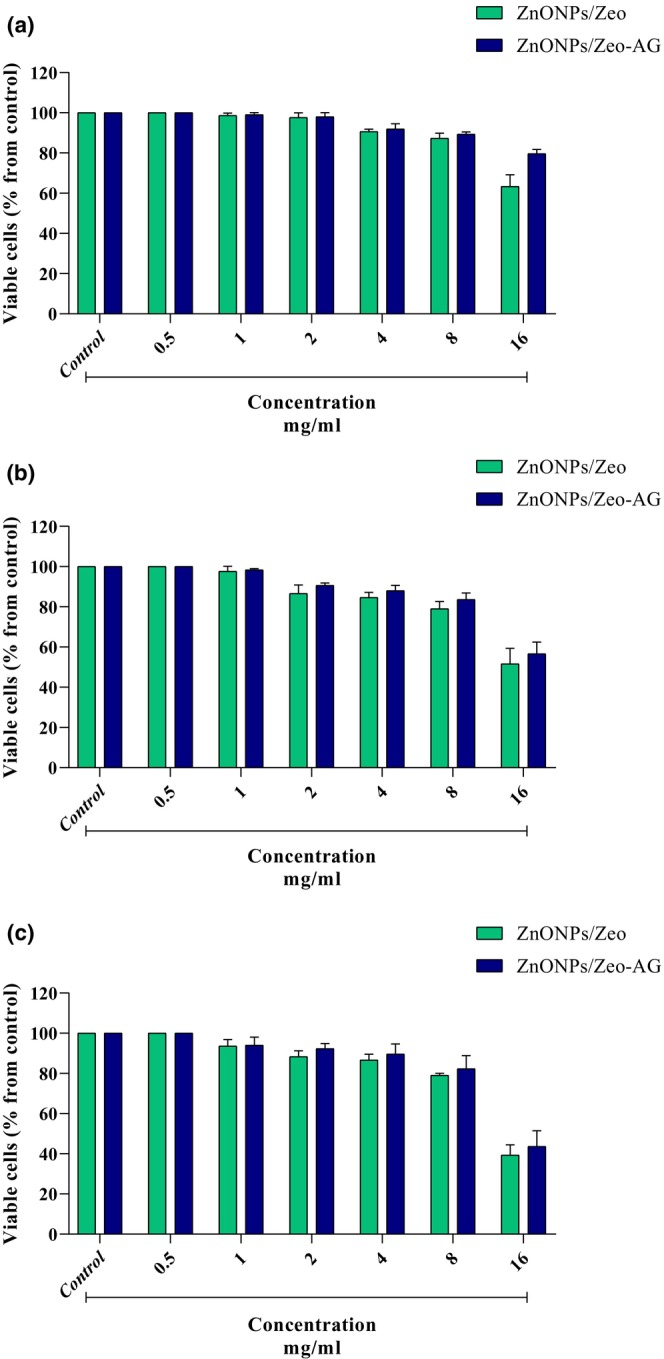
Effect of different concentrations of ZnONPs/Zeo and ZnONPs/Zeo‐AG on the viability of Caco‐2 cells at 24 h (a), 48 h (b), and 72 h (c).

The cytotoxicity properties of ZnONPs/Zeo and ZnONPs/Zeo‐AG on Caco‐2 cells (human colorectal adenocarcinoma cells) were evaluated by MTT assay. As shown in Figure 2, the following concentrations: 0.5–8 mg/mL of ZnONPs/Zeo and ZnONPs/Zeo‐AG caused a minor cytotoxic effect on Caco‐2 cells for 72 h in comparison with the control (100%) (*p* < .05). However, the survival rate was significantly diminished at a concentration of 16 mg/mL in comparison to the control group (untreated cells). These results suggest that these nanocomposites have the least cytotoxicity effect and good biocompatibility with the host. In addition, in the combination of *A. vera* gel with ZnONPs/Zeo, the cell viability of Caco‐2 cells was significantly higher in all concentrations compared to ZnONPs/Zeo.

### 3.3 Antibacterial effects of ZnONPs/Zeo and ZnONPs/Zeo‐AG

The susceptibility of two bacteria (*S. typhi* and *S. paratyphi* A) to zeolite, *A. vera* gel, ZnONPs/Zeo, and ZnONPs/Zeo‐AG was assessed using the microdilution method. Results showed the MIC of 8 and 4 mg/mL for ZnONPs/Zeo, in *S. typhi* and *S. paratyphi* A, respectively. On the other hand, the combination of ZnONPs/Zeo with *A. vera* gel inhibited the growth of *S. typhi* and *S. paratyphi* A at a lower concentration (MIC = 4 and 2 mg/mL, respectively). Also, none of the zeolite and *A. vera* compounds separately could inhibit the growth of the mentioned bacteria. The MBC test gave results equal to the MIC. That means, the MBC amount was 8 and 4 mg/mL for ZnONPs/Zeo in *S. typhi* and *S. paratyphi* A, respectively, and was 4 and 2 mg/mL for ZnONPs/Zeo‐AG in *S. typhi* and *S. paratyphi* A, respectively.

### 3.4 The preservative effect of nanocomposites on chicken fillets

**FIGURE 3:**
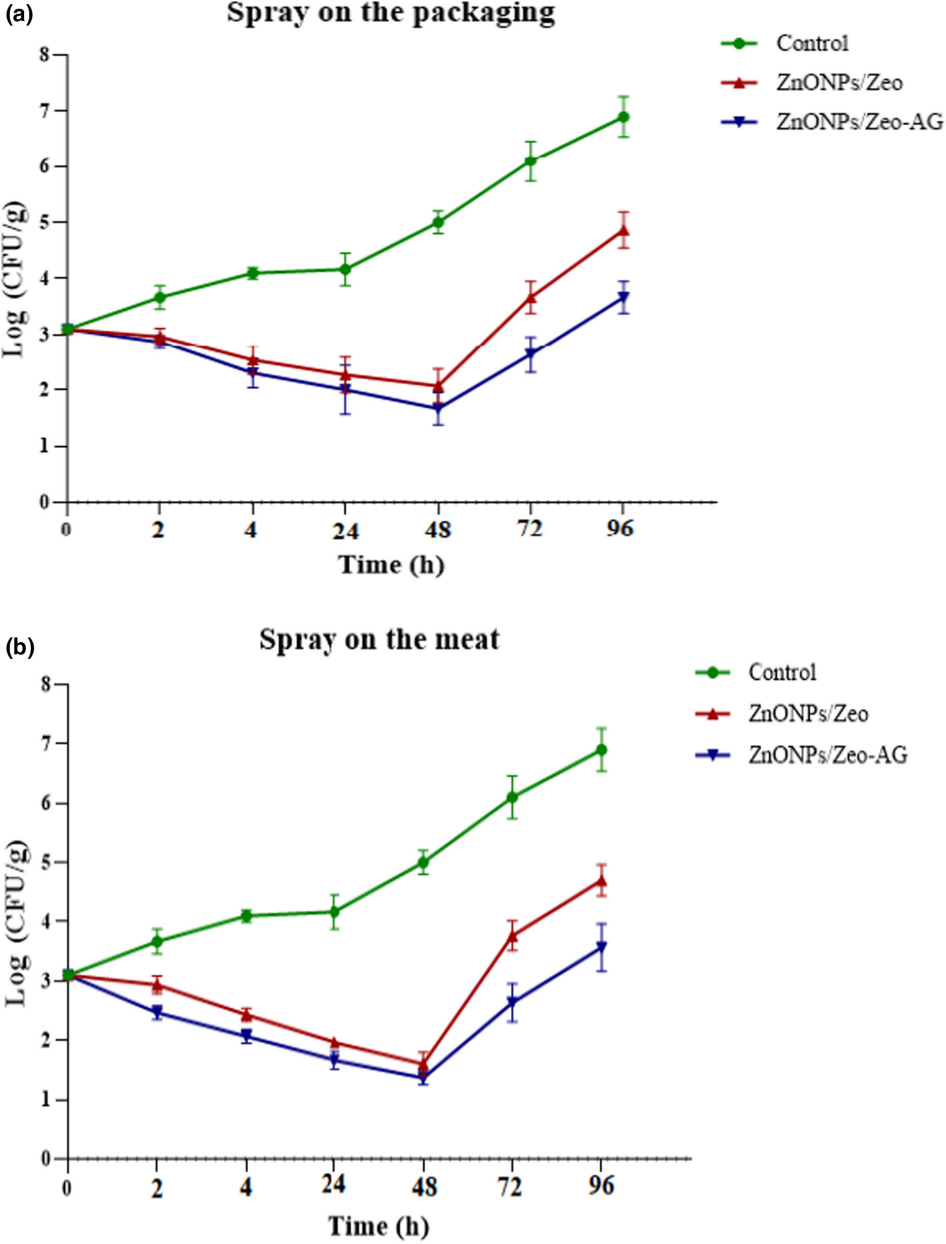
Colony counts of bacteria in chicken fillet samples were treated with the ZnONPs/Zeo and ZnONPs/Zeo‐AG by two methods: (a) spray on the packaging and (b) spray on the meat. Data are presented as the mean (−SD) of three independent biological replicates.

The number of viable microorganisms such as bacteria and fungi that chicken fillets contain at different time intervals (0, 2, 4, 24, 48, 72, and 96 h) and in the five examined groups was investigated. The findings showed that the number of bacterial colonies changes with time, so the highest shelf life of the samples was up to 48 h, and after that, an increase in the number of bacterial colonies was observed. It should be noted that in the method of spraying on chicken meat, the number of counted colonies was less compared with the spraying on the packaging. Also, the ZnONPs/Zeo‐AG caused a significant reduction in the number of bacterial colonies in both methods and was more effective in increasing the shelf life of chicken meat than the ZnONPs/Zeo (Figure 3).

## 4 DISCUSSION

Packaging is a key stage in the food industry; though its penetrable nature is the main defect in conventional packaging materials (Sharma et al., 2017). The researchers seek novel, cost‐effective, eco‐friendly, and biodegradable food packaging systems to preserve the quality of foods. It is an important factor driving the innovation of food packaging materials to be continuously evaluated (Sharma et al., 2017). The subsequent two types of materials are in attention: (1) inorganic and metal nanoparticles and (2) plant extract mixtures incorporated in biopolymers (Youssef et al., 2016; Yu et al., 2016).

Nanomaterials have unique properties compared with their bulk counterparts which have increased research on the synthesis, characterization, applications, and evaluation of these materials and have led to promoting the scientific progression and development of the agrifood area (Huang et al., 2018). Various reports have focused on the potential usage of nanomaterials as contributors to guarantee food quality, and rectify packaging problems (Huang et al., 2018).

The reason for gaps in understanding the toxicology of nanomaterials, and the expansion of their applications is associated with safety concerns. In the case of food, the primary phases of consumers' exposure are the movements of nanoparticles from packaging to food products (Störmer et al., 2017). Therefore, it is important to investigate the cytotoxic effects of these substances on human cells. In the present study, the cytotoxic effects of ZnONPs/Zeo and ZnONPs/Zeo‐AG on Caco‐2 cells were done using the MTT assay. As shown in Figure 2, in concentrations 0.5–8 mg/mL, ZnONPs/Zeo and ZnONPs/Zeo‐AG led to a slight cytotoxic effect on Caco‐2 cells for 72 h in comparison with the control (100%) (*p* < .05). Moreover, a significant cytotoxic effect was seen at the concentrations of 16 mg/mL of both substances in comparison with the negative control (100%) (*p* < .05). These results suggest that these nanocomposites have the least cytotoxicity effect and good biocompatibility with the host. Also, the cell viability of Caco‐2 cells was significantly higher in all ZnONPs/Zeo‐AG concentrations compared with ZnONPs/Zeo.

For assessing the susceptibility of pathogenic strains (*S. typhi* and *S. paratyphi* A), the microdilution method was used. The result showed that as the amount of both ZnONPs/Zeo and ZnONPs/Zeo‐AG increased, bacteria growth inhibition was observed. It is clear from growth inhibition that both of these materials have effective bactericidal activity. The higher impact was seen against *S. paratyphi* A, for which the MIC was 4 and 2 mg/mL for ZnONPs/Zeo, and ZnONPs/Zeo‐AG, respectively. Overall, the combination of ZnONPs/Zeo and *A. vera* gel had a superior inhibitory effect than the ZnONPs/Zeo alone. Moreover, experiments previously showed that ZnONPs/Zeo has significant antibiofilm activity against *Klebsiella pneumoniae* and *Staphylococcus aureus* strains in their sublethal concentrations (Partoazar et al., 2020, 2021).

Zeolite suspensions display no inhibitory effect, while the ZnONPs/Zeo suspensions for both tested bacteria presented antibacterial activity. These results are consistent with those found by Alswat et al. (2016). These antibacterial activities are because of different mechanisms. The first one is related to the ZnO nanoparticles size, which is the antibacterial activity enhancement with a reduction in the nanoparticle size (Alswat et al., 2016). The other mechanism is a result of the release of Zn ^2+^ ions to the medium comprising bacteria (Alswat et al., 2016).

Although antibacterial effects were reported for *A. vera* extract in previous studies (Arbab et al., 2021; Arsene et al., 2022), in our study, these materials separately did not have any antibacterial activity on the studied bacteria. This observation proposes that additional examination at concentrations higher than our tested concentrations should be done to measure their antimicrobial activity against *S. typhi* and *S. paratyphi* A. *A. vera* extract in combination with ZnONPs/Zeo displayed significant antibacterial activity against both tested bacterial strains. In the previous study by Ali et al. (2016), ZnONP‐AG showed differential antibacterial activity against Gram‐positive and Gram‐negative bacteria.

Microbial contamination is the most common disadvantage in the food industry, particularly in meat products, due to weakening quality and decreased shelf life of them. For this reason, in the current study, the effect of ZnONPs/Zeo‐AG on the shelf life of chicken was investigated. According to the results, for spraying on meat, 48 h after chicken fillet samples exposure to ZnONPs/Zeo‐AG and ZnONPs/Zeo, the number of bacteria reached 4.8, 1.5, and 1.8 log CFU/g in the control and ZnONPs/Zeo‐AG and ZnONPs/Zeo treatment samples, respectively (Figure 3). This number increased significantly after 48 h during the storage time, though the control had a quicker rate of increase in this parameter. This is due to bacterial resistance to the surrounding environment caused by the membrane and cell wall, with their components providing numerous penetrability pathways for NPs in bacteria (Lesniak et al., 2013). In Gram‐negatives, the cell wall's outer membrane consists of lipopolysaccharide (LPS), lipoproteins, and phospholipids. This causes resistance to the passage of substances. These structural attributes of the outer membrane prevent lipid peroxidation by ROS formed via ZnO NPs and decrease their susceptibility to ZnO (Kumar et al., 2017). At the end of the storage period, the number of bacteria was 7.2 log CFU/g in the control sample. The results also presented that after 48 h, a meaningfully higher count was seen in the control samples relative to the treatment sample sprayed with the ZnONPs/Zeo‐AG and ZnONPs/Zeo. Mostly, the nanocomposite containing ZnO NPs had a significant antimicrobial effect against the bacteria. A previous study suggests that NPs have superior antibacterial activity, similar to our study (Amjadi et al., 2019). Also, Esmaeili et al. (2021) previously reported that *A. vera* packaging can decrease decay by suppressing bacteria growth. This result approved the antibacterial activity of nanocomposites, particularly ZnONC/Zeo‐AG, and their potential for increasing the shelf life of chicken meat.

## 5 CONCLUSION

In the current research, an eco‐friendly, active nanocomposite containing ZnO NPs and *A. vera* was introduced. Considering the results of the microdilution test and bacterial count, the ZnO NPs displayed antibacterial activity against *S. typhi* and *S. paratyphi* A and it had no cytotoxic effect on Caco‐2 cells. In conclusion, antimicrobial active packaging comprising ZnO NPs and *A. vera* is a beneficial solution for preserving and improving the quality, safety, and shelf life of fresh meat products. It should be noted that it is better to conduct studies on migration assays and risk assessments of nanocomposite materials. Therefore, it allows the use of nanomaterials in the food packaging field.

## AUTHOR CONTRIBUTIONS

**Mohammad Mehdi Soltan Dallal:** Conceptualization (equal); project administration (equal); writing – review and editing (equal). **Samira Karimaei:** Formal analysis (equal); writing – original draft (equal); writing – review and editing (equal). **Maryam Hajighasem:** Investigation (equal). **Seyed Jamal Hashemi:** Formal analysis (equal); software (equal). **Abbas Rahimi Foroushani:** Formal analysis (equal). **Mahmoud Ghazi‐Khansari:** Formal analysis (equal); software (equal). **Alireza Partoazar:** Conceptualization (equal); supervision (equal).

## FUNDING INFORMATION

This study was part of a research project approved by the Food Microbiology Research Center, Tehran University of Medical Sciences, Tehran, Iran (Contract No. 46801), and has the ethics code IR.TUMS.VCR.REC.1399.209. We are grateful to the Vice‐Chancellor of Research at Tehran University of Medical Sciences who sponsored this research project.

## CONFLICT OF INTEREST STATEMENT

No competing interests to declare.

## Data Availability

All data that support the findings of this study are available in the manuscript.
